# EHMTI-0239. Effect of chronic paracetamol treatment on the csd-induced cgrp expression in the trigeminal ganglion

**DOI:** 10.1186/1129-2377-15-S1-F31

**Published:** 2014-09-18

**Authors:** W Yisarakun, W Supornsilpchai, C Chantong, T Thongtan, A Srikiatkhachorn, S Maneesri-le Grand

**Affiliations:** 1 Department of Pathology Faculty of Medicine, Chulalongkorn University, Bangkok, Thailand; 2 Department of Physiology Faculty of Dentistry, Chulalongkorn University, Bangkok, Thailand; 3 Department of Biochemistry Faculty of Medicine, Chulalongkorn University, Bangkok, Thailand; 4 Department of Physiology Faculty of Medicine, Chulalongkorn University, Bangkok, Thailand

## Introduction

Calcitonin gene related peptide (CGRP) is a neuropeptide which play an important role in the trigeminal nociception. Previous studies have demonstrated that chronic paracetamol (APAP) treatment resulted in the enhancement of trigeminal nociception. However, the effect of chronic APAP treatment on the CGRP has never been explored.

## Aim

To investigate the effects of chronic APAP treatment (30 days) on the CSD–induced CGRP expression in the trigeminal ganglion.

## Methods

Rats were divided into control, CSD only, APAP treatment only and APAP treatment with CSD groups. Once-daily injection of APAP at a dose of 200 mg/kg body weight was intraperitoneally injected into the APAP-treated groups for 30 days. CSD was induced by topical application of potassium chloride on the parietal cortex. The expression of CGRP was monitored by immunohistochemistry and the CGRP mRNA level was investigated by RT-PCR.

## Results

The induction of CSD caused an increase in the expression of CGRP in the trigeminal ganglion with a significantly higher in the number of CGRP-positive neuron than that observed in the control group. Interestingly, chronic APAP treatment in combination with or without CSD could significantly enhance the CGRP expression than that observed in CSD group. The results obtained from RT-PCR were in line with those obtained from immunohistochemical study. Chronic APAP treatment could significantly increase the CGRP mRNA level than that of control, especially in combination with CSD.

## Conclusion

Based on these results, it can be concluded that chronic APAP treatment can increase the CSD-induced CGRP expression in trigeminal ganglion.

No conflict of interest.

